# Nutrition, Growth, and Age at Puberty in Heifers

**DOI:** 10.3390/ani14192801

**Published:** 2024-09-27

**Authors:** Francesco Fantuz, Antonella Fatica, Elisabetta Salimei, Fausto Marcantoni, Luca Todini

**Affiliations:** 1 Scuola di Bioscienze e Medicina Veterinaria, Università degli Studi di Camerino, Via Gentile III da Varano, 62032 Camerino, Italy; luca.todini@unicam.it; 2 Dipartimento Agricoltura, Ambiente e Alimenti, Università degli Studi del Molise, Via Francesco de Sanctis 1, 86100 Campobasso, Italy; antonella.fatica@unimol.it (A.F.); salimei@unimol.it (E.S.); 3 Scuola di Scienze e Tecnologie, Università degli Studi di Camerino, Via Madonna delle Carceri snc, 62032 Camerino, Italy; fausto.marcantoni@unicam.it

**Keywords:** puberty, heifers, growth, nutrition, leptin, IGF-1

## Abstract

**Simple Summary:**

Age at puberty is a main factor affecting reproductive efficiency and, thus, livestock production. Besides species and breed, nutrition during growth plays a crucial role in the attainment of puberty. Controversial results are reported about the critical period during which heifers are more sensitive to the effects of nutrition on pubertal acceleration. Numerous papers are available about such topics, but they are characterized by very different experimental conditions (such as genetics, feeds, planes of nutrition, season and pasture quality, time windows, and parameters considered), making difficult the comparison among inconstant results by various trials. Several indicators during growth have been proposed as predictors of age at puberty, such as body weight gains, body fat reserves, and linear body measurements. Blood concentrations of hormones strictly linked to growth and reproduction have also been considered: higher Insulin-like Growth Factor-1 levels are reported to be related to earlier puberty, while leptin is not. More uniform experimental conditions should be encouraged for future research.

**Abstract:**

Puberty onset and age at first calving have a critical impact on livestock production for good reproductive efficiency of the herd and to reduce the duration of the non-productive stage of the growing heifer. Besides genetic factors, sexual maturation is also affected by environmental factors, such as nutrition, which can account for up to 20% of the observed variability. The rate of body weight gain during growth is considered the main variable influencing the age at puberty, dependent on planes of nutrition in growing animals during the prepubertal-to-pregnancy stage. This paper reviews current knowledge concerning nutrition management and attainment of puberty in heifers, considering the relevance of some indicators such as body measurements and hormones strictly linked to the growth and puberty process. Puberty onset is dependent on the acquisition of adequate subcutaneous adipose tissue mass, as it is the main source of the hormone leptin. Until a certain level, body condition score and age at puberty are negatively correlated, but beyond that, for fatter animals, such correlation is gradually lost. Age at puberty in heifers was reported to be negatively related to IGF-1. Future research should be planned considering the need to standardize the experimental animals and conditions.

## 1. Introduction

The timing of puberty onset and the age at first calving have a critical impact on livestock production [1,2]. Sexual maturation is controlled mainly by genetics but also by environmental factors, such as nutrition, which can account for up to 20% of the observed variability [3]. Nutrition management is based on different amounts of feed offered and/or on the inclusion of different feed materials in the diet, influencing its chemical composition in terms of energy and nutrient density.

It is well known that in growing animals, insufficient feed intake and/or an unbalanced diet may reduce growth performances and delay the onset of puberty [4,5], with a consequent reduction in the efficiency of the farms. On the other hand, increased nutrient intake during early development can advance puberty in females [6]. Body weight (BW) at weaning and even maternal nutrition during pregnancy can deeply impact female reproductive development [7,8], affecting the neuroendocrine axis development of the fetus and, thus, the future attainment of puberty [9].

Therefore, calf and heifer nutrition are strategic determinants since they affect animal physiology and development, considered crucial for future health and reproductive and productive performances [10,11]. In this regard, experimental findings and cost considerations in the dairy sector suggested raising dairy heifers calving at a younger age (22–24 months of age), provided that adequate frame size and organ development were achieved without negative effects on milk production [12,13,14]. To calve at 22–24 months of age at 82% of estimated mature BW, heifers need to conceive at 13–15 months of age at 55–60% of estimated mature BW [15], with puberty occurring at 43–55% of estimated mature BW in Holstein-Friesian animals [16]. Generally, within the seasonal beef production system mainly based on pasture, recommended guidelines have been to feed heifers to attain 60–65% of estimated mature BW at the beginning of the breeding season [17,18,19,20]. However, it has been reported that the development of lighter (lower ADG) beef heifers to 50–57% compared to heifers to 60–65% of estimated mature BW decreases costs without a negative impact on subsequent reproductive performances [21,22,23,24].

In recent years, the relationships of nutrition with neuroendocrine mechanisms related to the onset of puberty have been finely reviewed in bovine species [9,25,26,27,28]. The transition to puberty is allowed by the achievement of adequate body growth (also considering current body size relative to mature size) and, in particular, subcutaneous adipose tissue mass, this latter being transduced in proportional leptin secretion. Leptin does not act directly on GnRH neurons to activate the reproductive axis [29]. In the hypothalamic arcuate nucleus, leptin inhibits NPY (Neuropeptide Y) and stimulates POMC (proopiomelanocortin, the main component of the melanocortin system) neurons. NPY is an orexigenic peptide whose expression rises in conditions of negative energy balance, while projections of NPY neurons directly inhibit GnRH neurons’ activity and secretion. POMC neurons stimulate a subset of neurons (KNDy), which co-express neurokinin B, kisspeptin, and dynorphin as neurotransmitters. Neurokinin B (autocrine and paracrine) induces release of kisspeptin by KNDy with projections directly stimulating GnRH neurons. Dynorphin acts as negative feedback (autocrine and paracrine), hyperpolarizing and suppressing KNDy neuron activity. The sequential release of these three neurotransmitters by the KNDy neurons should be the basic mechanism responsible for the pulsatile nature of GnRH secretion [27,28,30,31,32]. On the other hand, it is known that excessive fat depots, including intrapelvic, can be responsible for dystocia and stillbirth in primiparous bovine [33]. Furthermore, excessive fat accumulation in mammary tissues before puberty can be responsible for decreased milk production in primiparous cows [34]. Moreover, too early onset of puberty in cattle may cause concerns about gamete quality and subsequent fertility [35].

Studies about the effects of nutritional management on puberty provided results that are often controversial and mainly linked to the species, breed, particular diets, and experimental conditions or feed availability. The aim of the present paper is to review current knowledge concerning nutrition management and the attainment of puberty in heifers. Particularly, the analysis of critical periods for the developing animals and the relevance of specific indicators, such as body measurements and hormones closely associated with growth and the pubertal process, i.e., leptin and Insulin-like Growth Factor-1 (IGF-1), provide a distinctive perspective on the subject matter.

## 2. Nutrient Intake and Growth

Nutrients (and energy) are required for both maintenance and growth by the calves and the heifers, and relationships among weight gain, age at puberty, and reproductive performance in heifers have been established so far [36]. Increased nutrient intake and growth rate can be used to positively affect the attainment of puberty and pregnancy in beef heifers [37]. Among morphological measures, BW (also as a proportion of estimated mature BW) can be considered the main factor influencing the herd puberty rate, according to Steele et al. [16]. In particular, the rate of Body Weight Gain (BWG) or Average Daily Gain (ADG) during growth is considered the most important variable related to age at puberty [38], affecting the time of puberty onset [9]. ADG is also the main variable that can be affected by planes of nutrition in heifers during the prepubertal-to-pregnancy stage. Elevated ADG and increased adiposity hasten the onset of puberty in various species, including cattle [39,40].

Holstein dairy heifers receiving diets with high (1.10 MJ ME and 20.9 g CP/100 g DM), medium (1.01 MJ ME and 18.1 g CP/100 g DM), and low (0.95 MJ ME and 13.5 g CP/100 g DM) energy and protein content to obtain ADG at 1.1, 0.8, and 0.5 kg/d starting from 100 kg liveweight, reached puberty at 9, 11, and 16 months of age, at 282, 282, and 316 kg BW, respectively [41]. According to Zanton and Heinrich [42,43], based on maximum milk production during the first lactation, the optimal ADG between 150 and 320 kg BW for Holstein heifers is 0.8 kg/d. Angus × Hereford heifers supplemented with different amounts of a mixture (24.6 g CP and 82 g TDN/100 g DM) of corn and dried distillers’ grains with solubles from weaning to first insemination to promote high ADG (0.8 kg/d), at the beginning of the breeding season, attained puberty in a greater proportion than heifers with moderate (0.6 kg/d) or low (0.4 kg/d; no supplementation) ADG [44]. In tropical dairy Criollo females, supplementary feeding (commercial mixed feed at 18 g CP/100 g as fed) increases ADG and decreases the age at puberty, compared to animals kept on pasture without supplementary concentrate [45], and similar results were also reported in crossbreed with *Bos indicus* beef heifers [46,47]. Crossbred heifers offered a higher feeding level (11.80 vs. 10.84 vs. 10.00 vs. 9.50 MJ/kg DM), based on ground corn and soybean meal supplement, showed a higher follicular diameter, and reached puberty at a younger age [48]. Analogously, Brahman (*Bos indicus*) heifers on improved pastures and fed higher amounts of concentrate (cotton seed meal and flaked barley) achieved puberty at an earlier age than heifers on standard pastures [49]. Nellore (*Bos indicus*) heifers at high energy intake (supplemented with mixed feed containing approx. 59% of wheat, 30% of corn, and 8% of soybean meal, ADG 0.9 kg/d) reached puberty earlier than those with low energy intake only fed forage (ADG 0.3 kg/d) [50]. Age at puberty resulted in lower than the breed average in heifers fed after weaning a feedlot diet (17 g CP/100 g DM) composed mainly of sorghum silage, corn, and soybean meal to ensure 0.7 kg/d of ADG [51]. Regardless of genetics, Nellore heifers fed an improved diet (feedlot grain-based, formulated to obtain ADG at 0.9 kg/d) had greater ADG, BW, and puberty rate compared with those at moderate nutrition (pasture grass-based diet plus 0.3 kg/100 kg BW of protein supplement, formulated to obtain ADG at 0.3 kg/d) [52]. Different results have been obtained in Buffalo species, as dairy buffalo heifers fed a feedlot diet (14–15 g CP and 0.80–0.88 feed unit for lactation/100 g DM, being one feed unit for lactation with 7.11 MJ of net energy for lactation) high in starch (21–22 g/100 g DM) showed higher Dry Matter Intake (DMI) and higher energy consumption than those kept at pasture (11–18 g CP and 0.60 feed unit for lactation/100 g DM), were heavier at puberty, but without any significant differences in the age at puberty [53]. The correlations found among BW, ADG, and age at puberty indicated that ADG seems to be important only for the buffalo heifers kept on pasture: grazing animals efficiently used the available feed resources (pasture and a limited hay supplementation) for their adequate development. In intensive rearing, once the dietary requirements for growth are fulfilled, spare nutrients are used mainly for fat mass deposition (as indicated by the higher Body Condition Score, BCS) without affecting age at puberty [53].

## 3. Critical Periods for the Impact of Nutrition

From the available literature, comparisons among studies are difficult, firstly due to the very large differences in experimental timing and quality of the diets. In addition to the relationships between metabolic homeostasis and puberty, very different results by different studies may have been due to differences in other environmental factors, including photoperiod [54,55], as reported in both *Bos taurus* [56] and *Bos indicus* [49,57] heifers. Furthermore, a reduction in voluntary DMI in hot environments is generally expected and observed [58]. In Brazil, Holstein calves born during the warmer months with higher THI (temperature humidity index) showed lower birth weight and lower BW at weaning and puberty compared to calves born in winter. The age at puberty was not directly influenced by the birth season, but it was influenced by weaning weight [59]. Several reports point to the actual time window in which improved nutrition and increased ADG are effective in anticipating puberty onset [25].

### 3.1. Prenatal Nutrition

Studies on rats indicate that early postnatal nutrition may be a determinant for the timing of puberty onset [60]. Moreover, metabolic developmental programming appears to start even earlier since, in rats, intrauterine growth retardation by maternal underfeeding results in delayed puberty onset [61,62]. However, results about age at puberty in Bovidae seem rather different from findings in rodents. Maia et al. [63] studied the effect of prenatal and postnatal nutrition in *Bos indicus*-influenced heifers. Cows were fed by the 6 months of gestation to achieve low (thin), moderate (normal), or high (obese) BCS, and their heifer offspring, weaned at 3.5 months of age, were fed post-weaning diets to obtain low (0.5/d) or high (1.0 kg/d) ADG until 8 months of age. Heifers from thin cows had lower BW at birth compared to heifers from obese cows, but BW was not different compared to cows with normal BCS, whereas it did not differ at weaning. However, it was not observed any effect of maternal diet nor its interaction with postnatal diet on age at puberty, but heifers on a high ADG postnatal diet reached puberty earlier than those on a low ADG postnatal diet. The authors concluded that in their experiment, postnatal dietary energy status (growth rate) was the only factor influencing age at puberty. Beef cows’ nutrition during late pregnancy and early lactation did not affect the age at puberty nor the percentage of heifers cycling before breeding in their offspring. Nonetheless, pregnancy rates and calving within the first 21 d of the heifers’ first calving season were higher for heifers from dams supplemented in late pregnancy (but not in early lactation) [7]. Analogously, dams’ nutrition during pregnancy had no effect on growth rates and age at puberty of the offspring, but increasing maternal nutrient intake during the third trimester can improve the first-service conception rates of daughters [64]. Several studies argue that while puberty is not affected by prenatal nutrition, lifetime heifer fertility may be compromised by maternal undernutrition [8,65]. On the other hand, maternal undernutrition reduced offspring postnatal gains at weaning, compromising metabolic status and follicle population during rearing, but did not impair performance in the first gestation and lactation periods of beef heifers [66]. In *Bos indicus*-influenced cattle, it was recently reported that maternal nutrition during the last two trimesters of gestation can have a modest effect on advancing puberty without any effect on fertility in the heifer offspring [63]. It is also postulated that *Bos indicus*-influenced females likely have an enhanced ability to adapt to extreme nutritional restrictions during gestation, thus protecting their offspring to a certain extent in comparison with *Bos taurus* females [27,28]. In general, in bovine, the ability to exert management control on nutritional impacts on puberty seems greater in the postnatal period compared to the prenatal period [27].

### 3.2. Preweaning Nutrition

Negative correlations between age at puberty and ADG before weaning (and not post-weaning) were reported [8,67]. Changes in the environment, management, and nutritional conditions that increase the animals’ pre-weaning weights and gains would reduce the age at puberty, anticipating sexual maturity [68]. For diets of dairy calves based on milk replacer plus starter feed, intensified feeding (milk replacer 30.6 g CP/100 g; starter feed 24.3 g CP/100 g) formulated to achieve ADG at 0.68 kg/d can be used to decrease age at first calving without negatively affecting future milk yield or economics, compared to conventional feeding (milk replacer 21.5 g CP/100 g DM; starter feed 19.9 g CP/100 g DM) formulated to achieve ADG at 0.45 kg/d [69]. Holstein calves fed milk replacer (140 g milk powder/kg) offered at 10% or 20%/kg BW did not show the difference in age at puberty, although the number and physiology of granulosa cells later in life were significantly affected [70]. The developmental plasticity could be restricted to the early stages of life [14]. In fact, calves are most efficient at converting nutrients to live-weight gain in early life [71] and, therefore, may benefit from high feeding rates in the pre-weaning period. Precocious pregnancy showed low correlations with post-weaning live weights and weight gains; therefore, the most adequate phase to evaluate the sexual precocity potential would be before weaning [68]. However, in beef heifers (weaned older than dairy ones), the effects of preweaning nutrition on puberty remain less clear than the impact of post-weaning nutrition since the developmental programming of the neuroendocrine axis should occur between 4 and 8 months of age [28]. The timing of supplementation seems determinant for the potential later effects. Earlier studies reported that providing starter feed ad libitum for up to 85 days prior to weaning increased weight gain (ADG) and advanced puberty [72], while from 68 to 118 days of age the supplementation did not affect metabolic hormones and age at puberty [73]. In *Bos indicus* heifers, puberty onset was not affected by starter feed supplementation at 110 to 205 days prior to weaning [51].

### 3.3. Precocious Weaning

Precocious weaning has been considered a valuable tool to anticipate growth and puberty. *Bos indicus* (Nellore) heifers early weaned (3.0 ± 0.1 months old and 84 ± 2 kg BW) and fed ad libitum a grain-based diet for high ADG (0.7–0.8 kg/d) post-weaning had a greater puberty rate than controls with low ADG (0.4–0.5 kg/d, obtained by offering 50% DM of the ad libitum DMI group at 19 months of age) [74]. Also, in Brahman beef crossbred heifers early weaned at 72 d [75] or at 14 wk [38], puberty was hastened when animals were fed high concentrate diets post-weaning. In fact, in this type of animal, age at weaning did not influence age at puberty if the body development was not different [76], and early weaned (118 ± 6 kg BW) heifers compared to normally weaned (183 ± 6 kg BW) cohorts showed worst puberty attainment and pregnancy rates, which were mainly dependent on the level of post-weaning protein supplementation [77]. Studies in *Bos taurus* seem to confirm such kind of results since no difference was found in age at puberty of Angus × Simmental heifers weaned 104 and 208 days old when animals were fed a high-energy diet (60% corn) compared to the control diet (30% corn) [78]. Weaning before the traditional age seems not essential for the effects of increased early growth rate to induce earlier puberty, as in early weaned heifers (112 d), the type of diet consumed from 126 to 196 d was a more important determinant of age at puberty than the type of diet consumed from 196 to 402 d [79].

However, in more precocious dairy breeds (Holstein Friesian and Jersey), Costigan et al. [80] compared growth and reproductive performances in heifers weaned 8 or 12 weeks old, and at the start of the mating season found higher rates of pubertal heifers for those weaned 12 weeks old, which also displayed better growth performances.

### 3.4. Post-Weaning Nutrition

The critical period during which heifers are more sensitive to the effects of nutrition on pubertal acceleration should be plausibly between 4–9 [26], 4–8 [27], and 4–7 [81] months of age. In Nellore heifers, prenatal and preweaning improved nutrition (concentrate supplementation) did not affect the age at puberty, whilst post-weaning feedlot diet (sorghum silage and concentrate on providing ADG at approx. 0.7 kg/d) was effective, compared to a diet based on pasture only [51,77]. Contrarily, Bruinjé et al. [82] did not observe differences in age at puberty due to either pre- or post-weaning different planes of nutrition in Holstein heifers. In beef heifers, controversial results were also reported. Higher post-weaning ADGs (obtained feeding a high-energy diet containing 60 g of ground corn/100 g DM) compared to a moderate energy diet (91 g of dehydrated alfalfa meal/100 g DM) were previously associated with earlier puberty onset [83], whilst no advantage was observed from higher post-weaning ADG obtained by Rodríguez-Sánchez et al. [84] supplementing heifers’ diet with mixed feed (44% corn and 22% barley) at 1 kg/100 kg BW, compared to heifers fed mixed feed at 0.4 kg/100 kg BW. Later, during the 60 days prior to breeding, a high compared to low starch diet (53 g vs. 37 g starch/100 g DM) may increase ADG and decrease the incidence of beef heifers at puberty that have had inadequate yearling BW [85]. Providing a high level of energy and protein supplementation (0.8 kg concentrate/100 kg BW, concentrate containing approx. 70 g of corn and 25 g of soybean meal/100 g DM) to Nellore heifers for approximately 100 d improves growth performance and increases the proportion of heifers that reach puberty before the mating season, associated with increased ADG [86].

As already mentioned, it is known that high-concentrate diets rich in rapidly fermentable carbohydrates may be responsible for excessive accumulation of fat at the mammary gland level, with decreased milk yield in subsequent lactations. On the other hand, excessive forage or NDF administration after weaning may decrease DMI and ADG, delaying the onset of puberty [14]. Regarding particular nutritional needs, Harvey et al. [87] highlighted the importance of trace mineral bioavailability: pregnant beef cows were supplemented with sulfate or organic complexed sources of Cu, Mn, Co, and Zn, and the puberty of their progeny heifers was hastened in the latter, despite similar ADG between treatments.

## 4. Effects of Nutrition on First Conception and Calving

Increased DMI and ADG after weaning advanced puberty and led to higher pregnancy rates: puberty rate at the beginning of the breeding season (13–15 months of age) was better in Angus × Hereford heifers fed supplement to promote high ADG (0.8 kg/d), in comparison with moderate (0.6 kg/d) or limited (0.4 kg/d) BW gains (87.5%, 62.5%, and 56.5%, respectively) [44]. Nellore heifers in post-weaning feedlots showed a higher puberty rate at 18 months of age than those on pasture (31.7 vs. 13.3%) [51]. *Bos indicus* heifers with improved nutrition from 11 months of age attained puberty within 23 months of age (11/11 vs. 1/11 with moderate nutrition) [49]. Daily supplementation of concentrate increased the percentage of pubertal crossbreed (Brangus) heifers on days 56 (57.6%), 89 (93%), and 168 (99.3%) of treatment (starting at 10–11 months of age) [47]. Puberty onset and subsequent fertility can be dependent on adequate subcutaneous adipose tissue, as reported in both *Bos indicus* [49] and *Bos taurus* [11] heifers. Subcutaneous fat is the main source of the metabolic hormone leptin, which signals to the central nervous system the amount of body energy reserves (see below Section 6 and Section 7.1). A relatively minor effect of BCS on pregnancy rates is also reported in *Bos indicus* [88,89] and crossbreed [90] heifers. It could be hypothesized that, in the rearing period, even moderate growth rates can be sufficient to reach the threshold weights recommended for first conception, and higher BW and fat deposition appear redundant and not associated with improved reproductive performances. In fact, beef heifers with higher DM intake at puberty resulted in younger and heavier BW, but there was no difference in pregnancy rate [83,91], as different post-weaning growth rates did not induce differences in age at conception and first calving [84]. Pregnancy rates were similar for Angus heifers, which developed to 55% or 62% of mature BW at puberty [92]. Analogously, crossbred beef heifers grazing winter pasture and placed in a feedlot showed greater ADG and pre-breeding BW in comparison with those grazing winter pasture and corn residue; nonetheless, the proportion of heifers attaining puberty before the breeding season and final pregnancy rates were similar [93]. On the other hand, more favorable body condition and physiological parameters (blood glucose and IGF-1) for heifers on improved nutrition were associated with a greater pregnancy rate to the first artificial insemination [52]. Finally, increasing maternal nutrient intake in beef cows during the third trimester, though not affecting growth rates and age at puberty of their daughters, can improve the first service conception rates [64].

Linked to nutrition management, the rearing system should be considered [94]. In dairy Buffalo heifers, age at puberty and conception rate were not affected by the rearing system (feedlot vs. pasture), which indeed caused different ADG and BW at puberty [95]. Studies on cattle seem to indicate pasture systems as favorable in comparison with intensive feedlots [96,97,98]. British crossbred heifers reared in feedlots had greater ADG but lower pregnancy rates compared with cohorts reared on range pastures [99]. Exercise is required for adequate reproductive function in cattle, as the lack of motor activity may alter puberty attainment and pregnancy rates despite adequate growth rates and final BW [100].

## 5. Body Measurements Indicators

In dairy cattle heifers, body measurements are proposed as reliable indicators among the tools for monitoring age at first breeding and calving [101]. Linear body measurements were found to be highly correlated with BW [80]. Heart girth (HG) can be influenced by different feeding levels both during preweaning and rearing periods, and it is strongly correlated with BW and BW gains [67]. Height at withers (WH) is a better indicator of animal development and size than BW, while HG (or chest depth) provides an indirect measure of the development of the gastrointestinal tract and liver. Different planes of nutrition in Holstein heifers influenced average BW and HG but had no effect on WH [102,103]. The literature review about the relationships between body measurements and age at puberty or first conception provides interesting, though inconclusive, results. In Nellore heifers, the age of puberty was not linked to BW, BCS, WH, and chest depth [104]. In Brahman crossbred heifers, offering concentrate had a positive linear effect on ADG and hip-width (HW) gain; however, the main determining factor for pregnancy success was the pre-mating BW, which was in turn affected by weaning weight or post-weaning supplementation [77]. Davis Rincker et al. [69] increased the energy and protein content in milk replacers for Holstein heifer calves, which at weaning had higher BW, WH, and HW in comparison with conventionally nursed pairs. Calves consuming the intensive diet had lower ADG during the final week before complete weaning and the early post-weaning period, and at the onset of puberty, were 31 d younger and 20 kg lighter. At first conception, they were 15 d younger and had a narrower HW [69]. Calves nursed ad libitum by whole milk and starter mixed feed had higher DM, energy, and protein intake, showed higher BW and skeletal parameters, and were 23 d younger at puberty than those fed restricted amounts of milk replacer and ad libitum starter mixed feed until weaning [105]. These effects of an intensified preweaning diet on timing puberty are suggested to be long-term since the differences in WH, HG, and HW among treatments, which were significant at weaning, became gradually smaller, and BW at puberty was not different [105]. Buffalo heifers bred in feedlot systems showing increased prepubertal gain and increased BW acquired significantly higher WH and HG at puberty, as compared to those bred on pasture systems, while body length and age at puberty were not different between the two groups [Terzano and Todini, unpublished results].

## 6. Body Fat Mass

Studies in humans and rodents have established that a critical threshold of body development and subcutaneous fat mass is required to trigger puberty [106]. In Bovidae heifers, subcutaneous fat accretion affects the puberty process [107,108,109]. Puberty onset and the response to first oestrus synchronization are dependent on the acquisition of subcutaneous adipose tissue that is sufficient to support reproductive function [104], as it is the main source of the hormone leptin [110,111]. Shamay et al. [105] observed that the age at which the BCS showed an accelerated increase, indicating enhanced fat deposition, coincided with puberty. Inverse relationships between age at puberty and adiposity state (measured as BCS or subcutaneous fat thickness by ultrasound scanning) have been reported. Chelikani et al. [41] observed that heifers fed post-weaning to obtain high (1.1 kg/d) or moderate (0.8 kg/d) ADG were younger and had higher BCS at puberty, compared to heifers fed to obtain low ADG (0.5 kg/d), whereas the BW and back-fat thickness were not different. Nonetheless, heifers on high and moderate ADG diets had higher body fat percentages compared to those on low ADG diets. Beef heifers fed to obtain high ADG (1.0 kg/d) starting from 8 until 16 months of age were younger, heavier, and had higher BCS and body fat percentage at puberty compared to heifers on a moderate ADG (0.6 kg/d) diet [112]. Until a certain level, BCS and age at puberty are negatively and linearly correlated, but beyond that, for fatter animals, such correlation is gradually lost [46]. In a recent study [104], age at puberty was not related to BCS, but more heifers with subcutaneous croup fat thickness (>3.4 mm) attained puberty, compared with heifers with lower values. In Nellore heifers, backfat thickness and subcutaneous rump fat thickness are significantly affected by both nutrition and genetics (selection) for early puberty [52]. Precocious pregnancy resulted in more subcutaneous fat thickness in Nellore heifers [68].

## 7. Endocrine Indicators

### 7.1. Leptin

Leptin is a protein hormone secreted primarily by adipose tissue, with major endocrine effects to regulate food intake and energy expenditure [113]. Body weight gains and adipose tissue accretion during juvenile development can enhance the synthesis and release of leptin [108], whose blood levels increase linearly as puberty approaches [114], signaling the central nervous system the availability of enough nutritional reserves to support the pubertal transition [81]. Leptin acts at the hypothalamic level, inhibiting NPY neurons (orexigenic and suppressive of GnRH neurons and thus secretion of gonadotropins) and stimulating POMC neurons. These latter, in turn, activate a subset of neurons (KNDy), which stimulate GnRH neurons by the release of kisspeptin [27,28,30,31,32].

Brahman heifers with improved nutrition, from approximately 16 months of age, had greater BW and BCS accompanied by higher circulating concentrations of insulin, leptin, IGF-1, and glucose [49]. Nutritional regimens that promote high ADG are accompanied by greater adiposity and increased circulating concentrations of the metabolic hormones leptin, insulin, and IGF-1 [26,115]. These endocrine signals play a critical role in puberty onset, suppressing the arcuate nucleus gene expression [115] and neuronal projections to GnRH neurons [116] of the inhibitory NPY while increasing gene expression and neuronal inputs of excitatory neuropeptides (e.g., POMC and α-Melanocortin) toward KNDy and GnRH neurons [27].

An increase in circulating concentrations of leptin in response to elevated BW gain was also reported in beef heifers [38], and following energy (starch from cracked corn) or protein (soybean meal) supplementation [100], closely associated with pubertal progression [108]. In Holstein heifers, leptin levels following different planes of nutrition seem to be related to the onset of puberty [82], and increased BW gain during juvenile development accelerated puberty, coincident with reciprocal changes in circulating concentrations of leptin and hypothalamic NPY release [39]. On the contrary, some papers reported no association between plasma leptin and diets in growing heifers [57]. Recently, Nellore heifers fed for high ADG since weaning to 3 months of age had greater serum leptin concentration than controls from 13 to 18 months old. Differently, serum IGF-1 concentration was greater at 5 and 7 months old and more associated with increasing DMI [74]. Despite early papers reporting accelerated onset of puberty in rodents treated with exogenous leptin [117], Block et al. [118] reported that plasma leptin in dairy cattle is regulated by nutrition in early postnatal life, but a sudden increase in plasma leptin is not required for the onset of puberty, which can occur over a wide range of plasma leptin concentrations. In heifers, exogenous leptin did not alter the secretion of LH, GH, insulin, and IGF-1 and had no effect on puberty [119]. The current and widely shared opinion is that leptin is a crucial hormone in determining the timing of puberty [108], but it does not trigger puberty, being a permissive yet critical signal for puberty onset [119,120]. A certain threshold of leptin levels is necessary for puberty attainment [121] to communicate at a central level of energy availability sufficient to support the transition to puberty.

Age at puberty in heifers was reported to be negatively related to IGF-1 [48,49,67] but not with leptin concentrations, which in turn was not influenced by feeding regimen nor related to weight gains or age at puberty [84]. Therefore, the function of leptin as an indicator of the growth and reproductive development of heifers can be considered secondary and less clear than that of IGF-1 [67].

### 7.2. IGF-1

Insulin-like Growth Factor-1 is mainly secreted by the liver and stimulated by Growth Hormone. Its synthesis and secretion depend on the nutritional status of the animal, and it acts as a metabolic indicator at several sites of the hypothalamus-pituitary-ovarian axis [122]. IGF-1 has been demonstrated to stimulate GnRH neuronal function in vitro and in vivo, both directly and through kisspeptin neurons. It is suggested that IGF-1 contributes to the timing of puberty by regulating the morphological development of GnRH neurons that is required for pubertal onset. Pituitary gonadotrophs have receptors for IGF-1, which enhances mitogenic activity and LH synthesis and/or secretion [123]. At the ovary, the role of the IGF-1 system in gonadotropin-induced folliculogenesis has been extensively investigated [124,125,126], as well as in ovarian steroidogenesis and corpus luteum function [127]. This hormone is deeply involved in the onset of puberty in cattle [49,67,121], and among all hormones and metabolites, it is considered the main factor correlated with reproductive performance and attainment of puberty in beef heifers [121]. Peaks of serum IGF-1 just before puberty onset have been recently reported in Nellore [74] and Buffalo [95] heifers. Leptin and IGF-1 often correlate with each other and are both strictly correlated to BCS and BW [128]. In growing buffalo heifers, plasma leptin showed significant positive correlations with BW and IGF-1 levels before puberty [95]. Elevated serum IGF-1 levels were related to improved nutrition in beef [67,73,84,129], in Nellore [130], as well as in Brahman [49] and crossbred [47] heifers. Faster body development with greater ADG was associated with higher IGF-1 levels, which in turn were related to earlier attainment of puberty [131]. Recently, it has been reported that improved nutrition only tended to raise IGF-1 concentrations, which were significantly affected by genetics, being higher in Nellore heifers selected for early age at puberty [52]. In Brahman × British crossbred heifers early weaned (72 d) and fed a high concentrate diet, the ADG in the 3 months post-weaning and the plasma IGF-1 at 3 and 6 months post-weaning explained approximately 34% of the variability in age at puberty [75]. Negative correlations of IGF-1 levels and age at puberty were not observed in dairy Buffalo heifers [95]. However, the nutritional-linked rise of IGF-1 levels, which was correlated with age at puberty, was observed at weaning (6 months of age) by Rodríguez-Sánchez et al. [67], or about one year before puberty (12–13 months of age) by Samadi et al. [49]. Kelly et al. [20] found that, in well-grown beef heifers, IGF-1 plasma concentration at 8 months of age is the stronger predictor of puberty age compared to morphological measures (including BW, linear body measurements, BCS, and ultrasonic measures of muscle depth and body fat) and other physiological measures (including blood leptin, adiponectin, and insulin, among others). Therefore, the potential effects of nutrition and associated systemic IGF-1 levels on timing the onset of puberty may take place at early stages of development [14].

Regarding fertility, Nellore beef heifers that became pregnant on the day of artificial insemination had higher IGF-1 concentrations compared to heifers that did not become pregnant, while no differences in BW, BCS, Growth Hormone, or leptin were recorded [132]. Furthermore, it was proposed that *Bos taurus* and *Bos indicus* cattle may differ in the effects of insulin and IGF-1 on reproductive function [122,132,133]. Breed [84] and intra-breed [57] genetic effects have been pointed out in this regard.

The effects of improved nutrition on leptin, IGF-1, and puberty reported in the literature are summarized in Table 1.

## 8. Conclusions

Puberty onset and age of first calving have a critical impact on livestock production. Besides genetic factors, sexual maturation is also affected by environmental factors, such as nutrition. According to recent reports, improved nutritional plans, i.e., increased energy and protein intake determining higher growth rate, can advance puberty in heifers. Considering the time window in which improved nutrition and increased growth rate are effective in advancing puberty onset, postnatal nutrition has, in general, a greater impact on advancing puberty compared to prenatal nutrition. Calves may benefit from improved nutrition during the preweaning period, but it is reported that the critical period during which growing females are more sensitive to the effects of nutrition on pubertal acceleration should be plausibly between 4 and 9 months of age. Morphological and physiological measurements were proposed as reliable indicators among the tools for monitoring age at first breeding and calving. Nonetheless, the wide literature available about such topics is characterized by very different experimental conditions (such as feeds, planes of nutrition, season and pasture quality, time windows, and variables considered), making difficult the comparison among inconstant results by various trials. Moreover, genetic differences between *Bos taurus* and *Bos indicus* can affect the responses to nutritional and environmental cues, as well as the roles and trends of metabolic hormones. Future research, including long-term studies, should be planned to take into account more standardized conditions in order to clarify the impact of nutrition during the different stages of growth.

## Figures and Tables

**Table 1:** Effect of improved nutrition (higher ADG) on blood concentration of leptin and IGF-1 and on attainment of puberty.

Species/Breeds	Ages	Experimental Diets	ADG kg/d	LEP	IGF-1	Puberty	Ref
Brahman	11–23 mo	Pasture plus conc (3.0–3.5 kg/animal × d vs. 1 kg/animal × d)	0.58 vs. 0.38	Higher	Higher	Earlier	[49]
Angus × Hereford × Brahman	4–8 mo	31/69 vs. 60/40 forage/conc ratio fed at higher or lower DMI	0.91 vs. 0.45	Higher	Not different	Not different	[38]
Angus × Hereford	From 226 d to 13 mo	Forage plus conc vs. forage	0.72–0.76 vs. 0.36	Higher	Higher	Higher puberty rate at the end of the trial	[100]
Holstein	Prewean 1–7 w Post-wean 11–25 w	10 vs. 5 L whole milk. 15/85 vs. 30/70 forage/conc	0.8 vs. 0.6 prewean 1.4 vs. 1.3 post-wean	Higher both pre and post-wean	nm	Not different. Earlier puberty is more likely with improved nutrition post-wean	[82]
Angus × Hereford × Brahman	From weaning (3.5 mo) to 11 mo	Same diet fed at higher or lower DMI	1.0 vs. 0.5	Higher	nm	Earlier	[39]
Nellore	9–28 mo	25/75 vs. 93/7 forage/conc ratio	0.7 vs. 0.3	Not different	Higher	Earlier	[57]
Nellore	3–7 mo 7–10 mo	Same diet fed at higher or lower DMI	0.79 vs. 0.45 0.70 vs. 0.50	Higher at 13–18 mo	Higher at 5–7 mo.	Higher puberty rate at 19 mo	[74]
Nellore × Hereford	From weaning until the puberty onset	34/66, 47/53, 78/22, 90/10 forage/conc ratio	1.15 vs. 0.94 vs. 0.73 vs. 0.54	nm	Higher at puberty	Earlier	[48]
Parda de Montaña	0–6 mo 6–15 mo	Milk plus conc ad libitum vs. milk Forage ad libitum plus 12 g vs. 6 g conc/kg BW	Target ADG 1.0 vs. 0.7	Not different	Higher at 3 and 6 mo	Earlier	[67]
Parda de Montaña, Pirenaica	6–15 mo	Forage ad libitum plus 10 g vs. 4 g conc/kg BW	0.8 vs. 0.6	Not different	Higher	Not different	[84]
Buffalo	8–21 mo	Feedlot vs. pasture	0.82 vs. 0.67	Higher	Higher	Not different	[95]
Angus × Hereford	From 9 mo to puberty	Different diets (different forage/conc ratio) fed at higher or lower DMI	Target ADG 1.36 vs. 0.68 vs. 0.23	nm	Higher	Earlier	[129]
Angus × Hereford	From 2 mo	Conc ad libitum for 50 d while nursing vs. no conc	0.75 vs. 0.49	Not different	Higher at the end of the treatment	Not different	[73]
Nellore	From 110 d of age pre weaning Post-weaning	Milk plus conc (6 g/kg BW) vs. milk for 112 d Forage plus conc (1 kg/animal × d) vs. forage for 112 d	0.76 vs. 0.62 Approx 0.3 vs. 0.07	nm	Higher	Not different	[130]
Angus × Brahman	From 1 y	Pasture plus conc at 1.75% vs. 1.25% of BW	0.71 vs. 0.65	nm	Higher	Higher puberty rate after 89 d of treatment	[47]
Nellore	From 13.8 mo	Conc-based diet vs. forage-based diet	0.97 vs. 0.23	nm	Higher	Higher pregnancy rate at FTAI	[52]

Abbreviations: ADG = average daily gain; DMI = dry matter intake; BW = body weight; Conc = concentrate; nm = not measured; FTAI = fixed time artificial insemination.
